# Concanamycins Are Key Contributors to the Virulence of the Potato Common Scab Pathogen *Streptomyces scabiei*

**DOI:** 10.1111/mpp.70175

**Published:** 2025-11-26

**Authors:** Corrie V. Vincent, Dawn R. D. Bignell

**Affiliations:** ^1^ Department of Biology Memorial University of Newfoundland St. John's Newfoundland and Labrador Canada

**Keywords:** common scab, concanamycins, phytotoxins, potato, *Streptomyces*, thaxtomin A

## Abstract

The soil‐dwelling bacterium *Streptomyces scabiei* is distributed worldwide and is the best‐characterised causative agent of common scab disease, which impacts potato crops and causes significant economic losses to growers. The principal pathogenicity factor responsible for common scab development is the phytotoxin thaxtomin A, which functions as a cellulose biosynthesis inhibitor in plants. *S. scabiei* also produces polyketide compounds belonging to the concanamycin family, which serve as inhibitors of eukaryotic vacuolar‐type ATPases and have been shown to exhibit phytotoxic activity against different plant species. It has been proposed that concanamycins contribute to the virulence of *S. scabiei,* but direct evidence of this has been lacking. Using constructed strains of *S. scabiei* that are either unable to produce concanamycins or produce elevated levels of the metabolites, we showed that concanamycins enhance the severity of disease symptoms induced by *S. scabiei* on radish seedlings and potato tuber tissue. We demonstrated that concanamycin production is controlled by two regulatory genes that are situated within the concanamycin biosynthetic gene cluster, and that production of concanamycins and thaxtomin A by *S. scabiei* is modulated by different nutritional signals. The concanamycin biosynthetic gene cluster is conserved in other common scab‐causing *Streptomyces* spp., suggesting that these metabolites may function as important virulence determinants in multiple phytopathogenic species. Overall, this study expands our understanding of the molecular factors that enable plant host colonisation and common scab disease development by *S. scabiei.*

## Graphical abstract

Production of concanamycins by the potato common scab pathogen *Streptomyces scabiei* is associated with an enhanced virulence phenotype and is controlled by genetic and environmental factors. Created using BioRender.

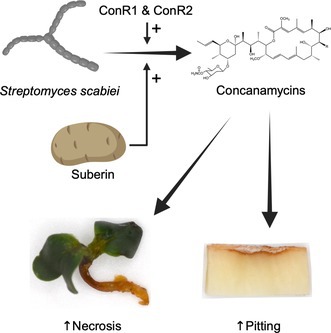

## 1 Introduction

Plant‐pathogenic species of the genus *Streptomyces* are the causative agents of common scab (CS) disease of potato ( *Solanum tuberosum* ). CS is characterised by the appearance of brown or dark‐coloured scab‐like lesions on the potato tuber surface. The symptoms of CS are highly variable; the lesions can be superficial, raised, or deep pitted, and can vary in size and shape (Loria et al. 1997). The symptom development is dependent on many factors, including environmental conditions, the susceptibility of the potato cultivar, and pathogen virulence (Clarke et al. 2019; Dees and Wanner 2012). Although potato is the most economically important host, CS can also affect other root and tuber crops, including radish, beet, and carrot (Clarke et al. 2022; Goyer and Beaulieu 1997). CS occurs throughout potato‐growing regions worldwide, and the presence of scab lesions reduces the market value of affected tubers, leading to significant financial losses for growers (Dees and Wanner 2012; Hill and Lazarovits 2005). CS is notoriously difficult to control, with no consistently reliable management strategies currently available (Biessy and Filion 2022; Dees and Wanner 2012).

*Streptomyces scabiei* (syn. *S. scabies*) is the first described and best characterised CS‐causing pathogen and is distributed worldwide (Lambert and Loria 1989). In addition to *S. scabiei*, more than 20 other phytopathogenic *Streptomyces* spp. have been described, including the CS pathogen *S. turgidiscabies* and the acid scab pathogen *S. acidiscabies* (Vincent and Bignell 2024). The principal pathogenicity determinant produced by most scab pathogens is the diketopiperazine phytotoxin thaxtomin A (ThxA), which functions as a cellulose biosynthesis inhibitor (Li et al. 2019). ThxA induces necrosis of excised potato tuber tissue (Loria et al. 2006), and treatment of monocot and dicot seedlings with the toxin results in root and shoot stunting and thickening, and root necrosis (Leiner et al. 1996).

Additional known virulence factors produced by *S. scabiei* and other phytopathogenic *Streptomyces* include the secreted necrogenic protein Nec1 and the plant hormone indole‐3‐acetic acid (Li et al. 2019). *S. scabiei* also produces *N‐*coronafacoyl‐L‐isoleucine (CFA‐Ile), which is proposed to function as a mimic of the plant hormone jasmonoyl‐L‐isoleucine (Bignell et al. 2018). CFA‐Ile has been shown to contribute to the virulence of *S. scabiei* and has a synergistic effect on symptom severity with ThxA (Bignell, Seipke, et al. 2010; Cheng et al. 2019). Unlike ThxA, CFA‐Ile production is not widespread among phytopathogenic *Streptomyces* (Bignell et al. 2018).

**FIGURE 1:**
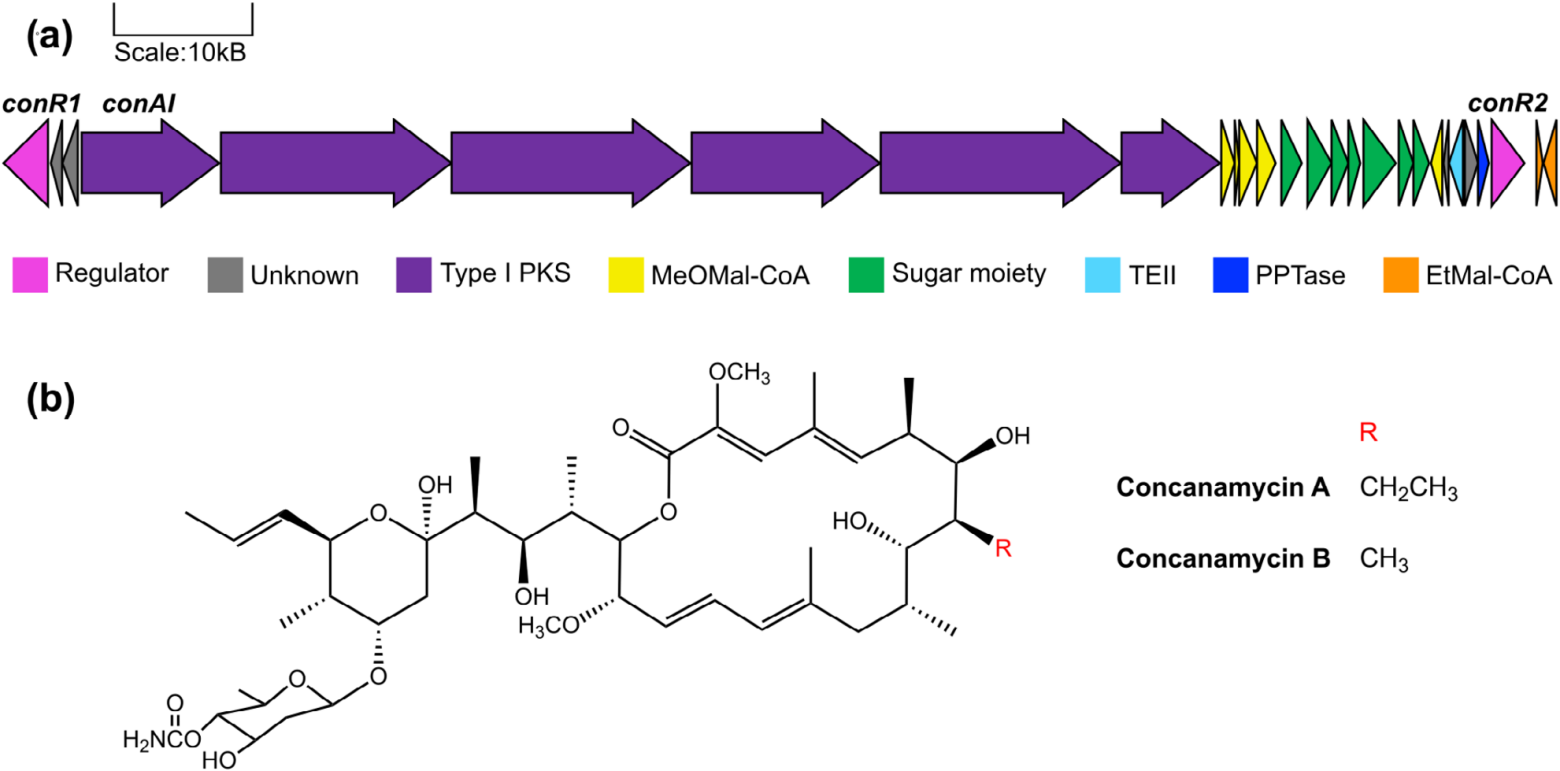
(a) Organisation of the concanamycin biosynthetic gene cluster in *Streptomyces scabiei* 87‐22. Genes are represented by the block arrows, with the direction of transcription indicated by the direction of the arrow. The genes subjected to deletion in this study (*conAI, conR1, conR2*) are indicated. Type I PKS = Type I polyketide synthase, MeOMal‐CoA = methoxymalonyl‐CoA biosynthesis, Sugar moiety = biosynthesis and attachment of 4′‐O‐carbamoyl‐2′‐deoxyrhamnose moiety, TEII = type II thioesterase, PPTase = phosphopantetheinyl transferase, EtMal‐CoA = ethylmalonyl‐CoA biosynthesis. The gene cluster diagram was made in Gene Graphics (Harrison et al. 2018). (b) Chemical structure of concanamycins A and B. The structure was drawn in ChemDraw 20.1.

The concanamycins are 18‐membered macrolides that were first isolated from the nonpathogenic species *Streptomyces diastatochromogenes* S‐45 (Kinashi et al. 1984). The production of concanamycins A and B (CMA, CMB; Figure 1) by *S. scabiei* was first reported nearly 30 years ago (Natsume et al. 1996). The concanamycins are effective inhibitors of vacuolar‐type ATPases (V‐ATPases) and possess antifungal, phytotoxic, and cytotoxic activities (Dröse and Altendorf 1997; Natsume et al. 1996; Westley et al. 1984). V‐ATPases are ubiquitous in eukaryotic cells and are the dominant proton pump in plant cells (Seidel 2022; Vasanthakumar and Rubinstein 2020). In plant cells, V‐ATPase inhibition by CMA has been associated with vacuolation and swelling of the Golgi apparatus (Robinson et al. 2004), accumulation of secretory and endocytic cargos in trans‐Golgi network‐derived structures (Dettmer et al. 2006), accumulation of autophagic bodies in the central vacuole (Yano et al. 2015), aggregation of endosomal compartments, and blocked endosomal trafficking (Zhou et al. 2016).

CMA and CMB have been shown to inhibit the growth of monocot and dicot seedlings (Natsume et al. 2005), and CMA produces weak necrotic lesions on excised potato tuber slices (Natsume et al. 2017). Co‐administration of ThxA and CMA to potato tuber slices showed a synergistic effect on thaxtomin toxicity (Natsume et al. 2017). It was observed that *S. scabiei*, which produces both ThxA and concanamycins, tends to produce more deep‐pitted scab lesions, whereas *S. acidiscabies* , which produces ThxA but not concanamycins, tends to produce flat or raised lesions (Natsume et al. 2017). This suggests that concanamycins may contribute to the formation of more deep‐pitted CS lesions, though this has remained speculative as there are other differences in the known or predicted virulence factors produced by *S. scabiei* and *S. acidiscabies* besides concanamycins.

Here, we demonstrate through mutational studies that the concanamycins play a key role in *S. scabiei–*plant interactions by enhancing the virulence of *S. scabiei* . We show that loss of concanamycin production reduces the severity of disease symptoms induced by *S. scabiei* during colonisation of radish seedlings and excised potato tuber tissue, whereas overproduction of these molecules augments the severity of disease symptoms. We also show that two predicted regulatory genes within the *S. scabiei* concanamycin biosynthetic gene cluster function as positive activators of concanamycin production in *S. scabiei*, and that the production of concanamycins and ThxA is differentially affected by nutritional signals. Importantly, the concanamycin biosynthetic gene cluster (BGC) is conserved in multiple phytopathogenic *Streptomyces* spp., suggesting that concanamycins may function as virulence factors in several plant pathosystems.

## 2 Results and Discussion

### 2.1 The Concanamycin BGC Is Conserved in Several Different Scab‐Causing *Streptomyces* Species

The concanamycin BGC was first described in the nonpathogen *S. neyagawaensis* (Haydock et al. 2005), and a homologous cluster is present in the genome of *S. scabiei* 87‐22 (Liu et al. 2021) (Figure 1). The polyketide backbone of concanamycin is biosynthesised by a multimodular (type I) polyketide synthase (PKS) encoded across six genes, *conAI–conAVI* (Haydock et al. 2005). The BGC also contains genes required for the provision of methoxymalonyl‐ and ethylmalonyl‐CoA extender units, biosynthesis, and attachment of the 4′‐O‐carbamoyl‐2′‐deoxyrhamnose moiety, a phosphopantetheinyl transferase (PPTase) for post‐translational activation of the PKS, a type II thioesterase for proofreading PKS activity, and two cluster‐situated transcriptional regulators (CSRs) (Haydock et al. 2005).

**FIGURE 2:**
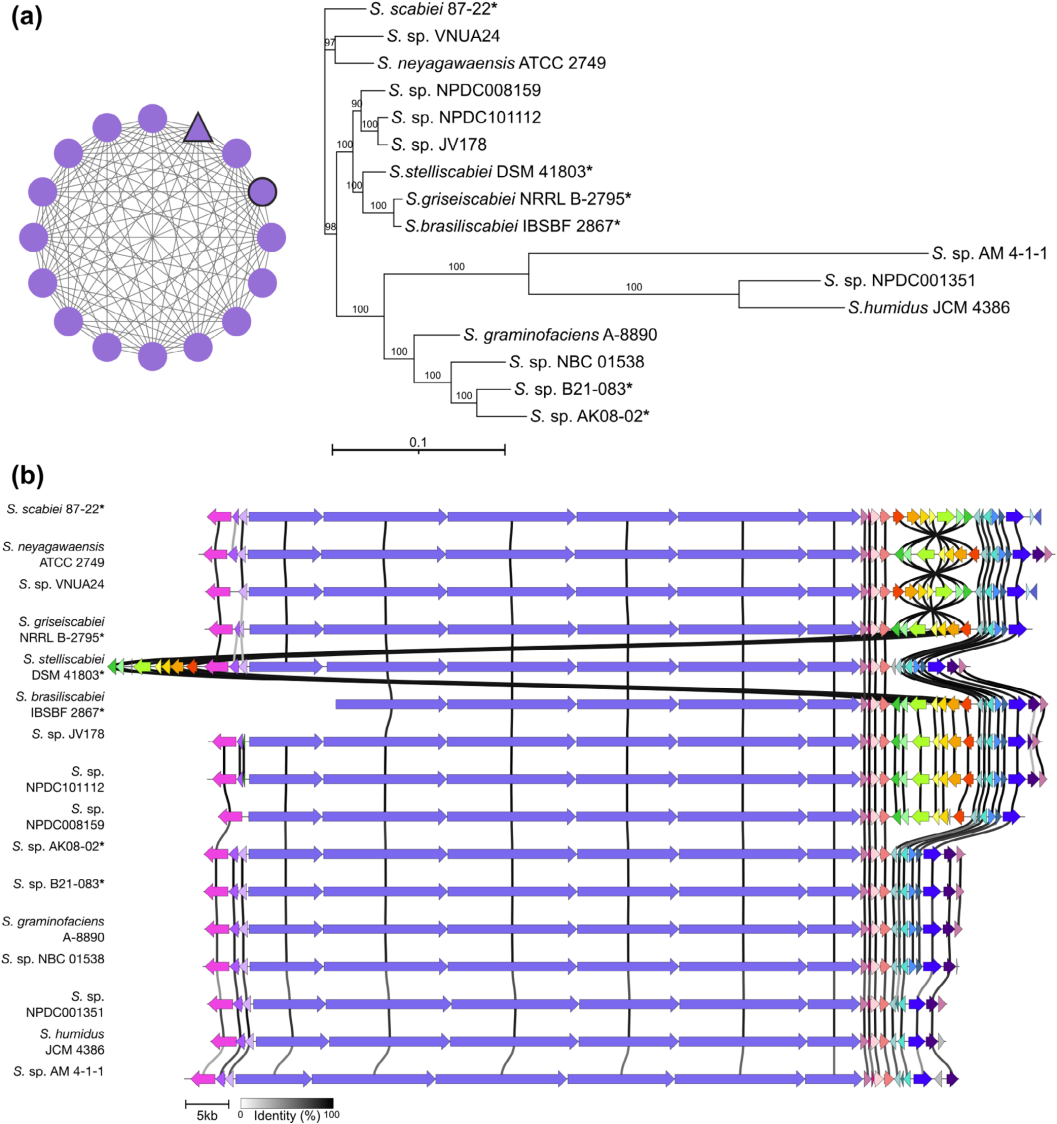
Analysis of concanamycin biosynthetic gene clusters (BGCs) from *Streptomyces* species. (a) Similarity network of *S. scabiei* 87‐22 concanamycin BGC and BGCs from other *Streptomyces* species (left) and their evolutionary relationships within the gene cluster family (right). The triangular node represents *S. scabiei* 87‐22; the circular node with thick black outline represents the known *S. neyagawaensis* BGC from MiBIG database. Bootstrap values ≥ 50% are shown at the respective branch points and are based on 1000 repetitions. (b) Alignment of the *S. scabiei* 87‐22 concanamycin BGC with the known concanamycin BGC from *S. neyagawaensis* ATCC 27449 and other BGCs within the same gene cluster family. Genes coloured the same belong to the same functional group, and homologues are linked by shaded areas that indicate the % identity. The strains that are reported plant pathogens are indicated with an asterisk (*).

The BiG‐SCAPE (biosynthetic gene similarity clustering and prospecting engine) tool (Navarro‐Muñoz et al. 2020) was used to investigate the prevalence and conservation of concanamycin‐like BGCs among *Streptomyces* spp. BGCs that grouped into the same gene cluster family as the *S. scabiei* concanamycin BGC were identified in several different species and strains, including other known CS pathogens such as *S. brasiliscabiei* (Corrêa et al. 2021), *S. griseiscabiei* (Nguyen et al. 2022), and *S. stelliscabiei* (Bouchek‐Mechiche et al. 2000) (Figure 2a). Production of CMA by *S. stelliscabiei* and *S. griseiscabiei* was recently demonstrated by Pereira et al. (2025). In some genomes, the cluster appears to be incomplete (Figure 2b), but this is likely due to the genome sequences being unassembled. For example, in *S. brasiliscabiei* and *S. griseiscabiei*, homologues of genes missing from the concanamycin‐like BGC were detected on a separate contig.

When comparing the architecture of the different concanamycin‐like BGCs, the deoxysugar subcluster is the only region that appears to frequently vary in its presence, location and orientation (Figure 2b). This subcluster consists of seven genes that are required for the biosynthesis and attachment of the 4′‐O‐carbamoyl‐2′‐deoxyrhamnose to concanamycin F (CMF) to produce CMA (Haydock et al. 2005). Compared to *S. neyagawaensis*, the orientation of the deoxysugar subcluster in the *S. scabiei* concanamycin BGC is reversed, whereas the same orientation is observed in *S. stelliscabiei*, except that the genes are located at the opposite end of the BGC (Figure 2b). Two recently reported CS pathogens, *Streptomyces* sp. AK08‐02 (Weisberg et al. 2023) and *Streptomyces* sp. B21‐083 (Biessy et al. 2024), both contain a concanamycin‐like BGC that lacks the deoxysugar subcluster (Figure 2b) and thus are predicted to be able to produce CMF but not CMA (or CMB). Notably, CMF possesses similar V‐ATPase inhibitory activity as CMA and CMB (Woo et al. 1992).

The virustomycin A BGC from *S. graminofaciens* A‐8890 was included in the analysis as it was previously shown that CMF is modified with fumarate and C_5_N appendages to produce virustomycin A (Kudo et al. 2023), and thus the BGC can be considered a concanamycin‐like BGC. The virustomycin A BGC lacks the deoxysugar subcluster, and three additional genes required for the biosynthesis of the C_5_N unit of virustomycin A are encoded in a separate region of the genome (Kudo et al. 2023). Homologues of such genes were not detected in the genome of *S. scabiei* and the other phytopathogenic *Streptomyces* spp., and so these species are not expected to be able to produce virustomycin A.

### 2.2 Differential Production of Thaxtomin A and Concanamycins by *S. scabiei* in Response to Nutritional Signals

**FIGURE 3:**
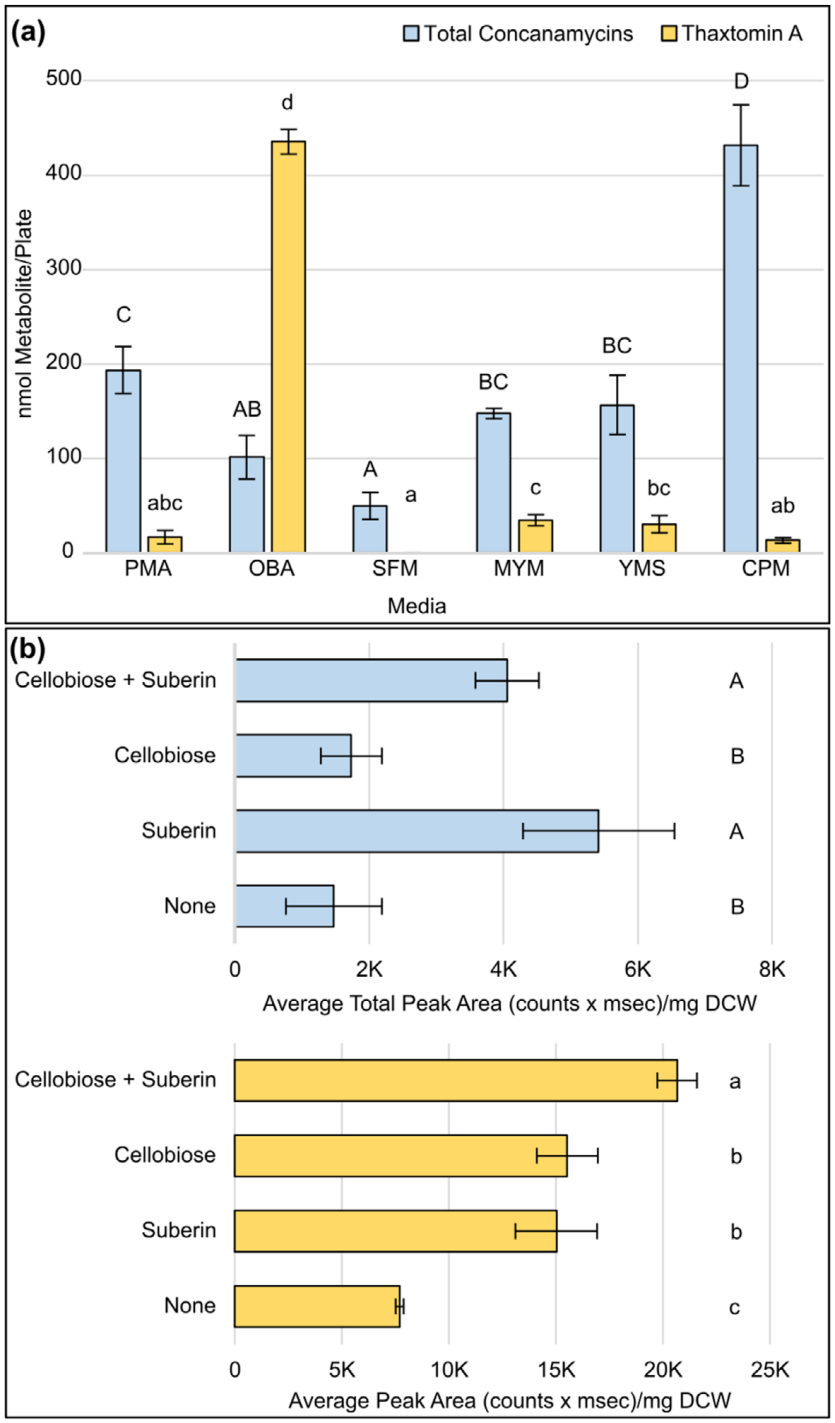
(a) Production of concanamycins and ThxA by wild‐type *Streptomyces scabiei* cultured on six different solid media. Each bar shows the mean metabolite production level (*n* = 3), and error bars represent the standard deviation from the mean. Means with different letters (A–D for concanamycins; a–d for ThxA) were determined to be significantly different (*p* < 0.05). (b) Production of concanamycins (blue bars) and ThxA (orange bars) by wild‐type *S. scabiei* cultured on ISP4 (International *Streptomyces* Project 4) medium supplemented with 0.5% wt/vol cellobiose and/or 0.1% wt/vol suberin, or without supplementation. Each bar shows the mean metabolite production level (*n* = 3), and error bars represent the standard deviation from the mean. Means with different letters (A–D for concanamycins; a–d for ThxA) were determined to be significantly different (*p* < 0.05). CPM, concanamycin production medium. DCW, dry cell weight; MYM, maltose‐yeast extract‐malt extract; OBA, oat bran agar; PMA, potato mash agar; SFM, soy flour mannitol; YMS, yeast extract‐malt extract‐starch.

ThxA production by *S. scabiei* and other scab pathogens occurs primarily in plant extract‐containing media (Loria et al. 1995; Beauséjour et al. 1999) and is induced in particular by plant‐derived cello‐oligosaccharides (cellobiose and cellotriose) and suberin (Johnson et al. 2007; Lerat et al. 2010). In testing six different solid media for the production of ThxA and concanamycins by *S. scabiei* 87‐22 (Figure 3a), we found that, as expected, oat bran agar (OBA) was the only medium that could support high levels of ThxA production. This medium is rich in cello‐oligosaccharides, which cause de‐repression of the ThxA biosynthetic genes by the cellulose utilisation transcriptional repressor CebR (Francis et al. 2015; Jourdan et al. 2016). Production of concanamycins exceeded that of ThxA in all of the tested media except OBA, with the highest production levels occurring in concanamycin production medium (CPM) (Figure 3a). In this medium, both CMA and CMB were detected, with CMB routinely being the predominant derivative in the acetonitrile culture extracts (Figure S1). Other concanamycin derivatives that were detected include the aglycone precursor of CMA (CMF), the anhydroaglycone derivatives of CMA and CMB (concanamycins H [CMH] and G [CMG], respectively), and the 21‐*O*‐methyl derivatives of CMA and CMB (Figure S1; Table S1). The formation of the latter two metabolites in methanol extracts has been previously described (Kinashi et al. 1981; Kinashi et al. 1984; Liu et al. 2021; Woo et al. 1992) and both metabolites were absent or reduced in extracts prepared using acetonitrile (ACN) (Figure S1). This suggests that the *O*‐methyl CMA and CMB derivatives mainly represent artefacts of the extraction process rather than natural products generated by *S. scabiei*. Although CMB was the main concanamycin detected in the CPM ACN extracts, CMA was the predominant derivative detected in the ACN culture extracts for all the other media tested except potato mash agar (PMA), where CMA and CMB were detected in roughly equivalent amounts (data not shown).

Although previous analyses of concanamycin production by *Streptomyces* spp. have primarily been conducted using liquid cultures (Haydock et al. 2005; Kinashi et al. 1984; Natsume et al. 1996; Woo et al. 1992), the majority of *Streptomyces* spp., including *S. scabiei* , are only able to undergo morphological development on solid media, and this process is typically coordinated with the production of specialised metabolites (McCormick and Flärdh 2012; Van Wezel and McDowall, van Wezel and McDowall 2011). We found that *S. scabiei* produces higher levels of concanamycins when cultured on solid CPM versus liquid CPM (Figure S2), suggesting that the ability to undergo morphological development is important for the production of these metabolites.

Given that cello‐oligosaccharides and suberin are known inducers of ThxA production in *S. scabiei*, we investigated whether concanamycin production is also induced or enhanced by the presence of cellobiose and/or suberin in the culture medium. The addition of either 0.5% wt/vol cellobiose or 0.1% wt/vol suberin to ISP4 (International *Streptomyces* Project Medium 4) solid medium caused a significant increase in ThxA production compared to the medium lacking either amendment, and production was even greater in medium supplemented with both compounds (Figure 3b), a finding that is consistent with previous research from Prof. Carole Beaulieu's group (Lerat et al. 2010). Suberin was also found to increase production of total concanamycins in the ISP4 medium (Figure 3b), though production levels were generally very low in this medium. Suberin is a recalcitrant plant polymer that is found in the potato periderm, and it has been shown to induce the production of specialised metabolites by different *Streptomyces* spp., possibly by inducing morphological development (Lerat et al. 2011). The addition of cellobiose, on the other hand, had no significant impact on production of the concanamycins by *S. scabiei* (Figure 3b). This, together with the fact that OBA did not support high levels of concanamycin production (Figure 3a), suggests that cello‐oligosaccharides do not play an important role in controlling the production of concanamycins, a finding supported by the absence of predicted CebR binding sites within the concanamycin BGC (Deflandre et al. 2022). However, Deflandre et al. (2022) previously reported that CMA/CMB production was induced by the addition of either cellobiose or cellotriose to a minimal culture medium, and although they used less of these inducers (2.5 mM or 0.09% wt/vol) in their culture medium compared to the 0.5% wt/vol of cellobiose used here, we also saw no significant induction of concanamycin production with lower cellobiose concentrations (0.1% and 0.25% wt/vol; data not shown). Possibly, other differences in culture conditions between our study and theirs may account for the discrepancy in the role of cellobiose as an inducer of concanamycin production. Notably, Planckaert et al. (2018) also reported that CMA production is not induced in wild‐type (WT) *S. scabiei* when cellobiose is added to ISP4 medium, but they did observe elevated production when the *cebR* gene is deleted. Thus, the role of cello‐oligosaccharides and CebR in controlling the production of concanamycins requires further investigation. Overall, our results show that ThxA and concanamycin production by *S. scabiei* is mediated by different nutritional signals, and potato‐derived suberin can enhance the production of both phytotoxins under laboratory conditions.

### 2.3 ConR1 and ConR2 Are Positive Activators of Concanamycin Biosynthesis in *S. scabiei*

The concanamycin BGC contains two genes with predicted regulatory function, *conR1* (*scab83841*) and *conR2* (*scab84101*) (Haydock et al. 2005; Figure 1). ConR1 is a member of the large‐ATP binding regulators of the LuxR (LAL) family, which are relatively common in *Streptomyces* BGCs and typically function as positive regulators of specialised metabolite production (Romero‐Rodríguez et al. 2015; Wilson et al. 2001). ConR2 is a member of the *Streptomyces* antibiotic regulatory protein (SARP) family, which is among the most common and best characterised types of CSR in *Streptomyces* BGCs and typically functions as a positive regulator (Romero‐Rodríguez et al. 2015; Yan and Xia 2024). SARPs most often function at the end of the signal transduction cascade, directly activating transcription of biosynthetic genes. Homologues of both *conR1* and *conR2* are conserved in the concanamycin and concanamycin‐like BGCs (including the virustomycin A BGC) identified in other *Streptomyces* spp. (Figure 2b) (Pereira et al. 2025).

**FIGURE 4:**
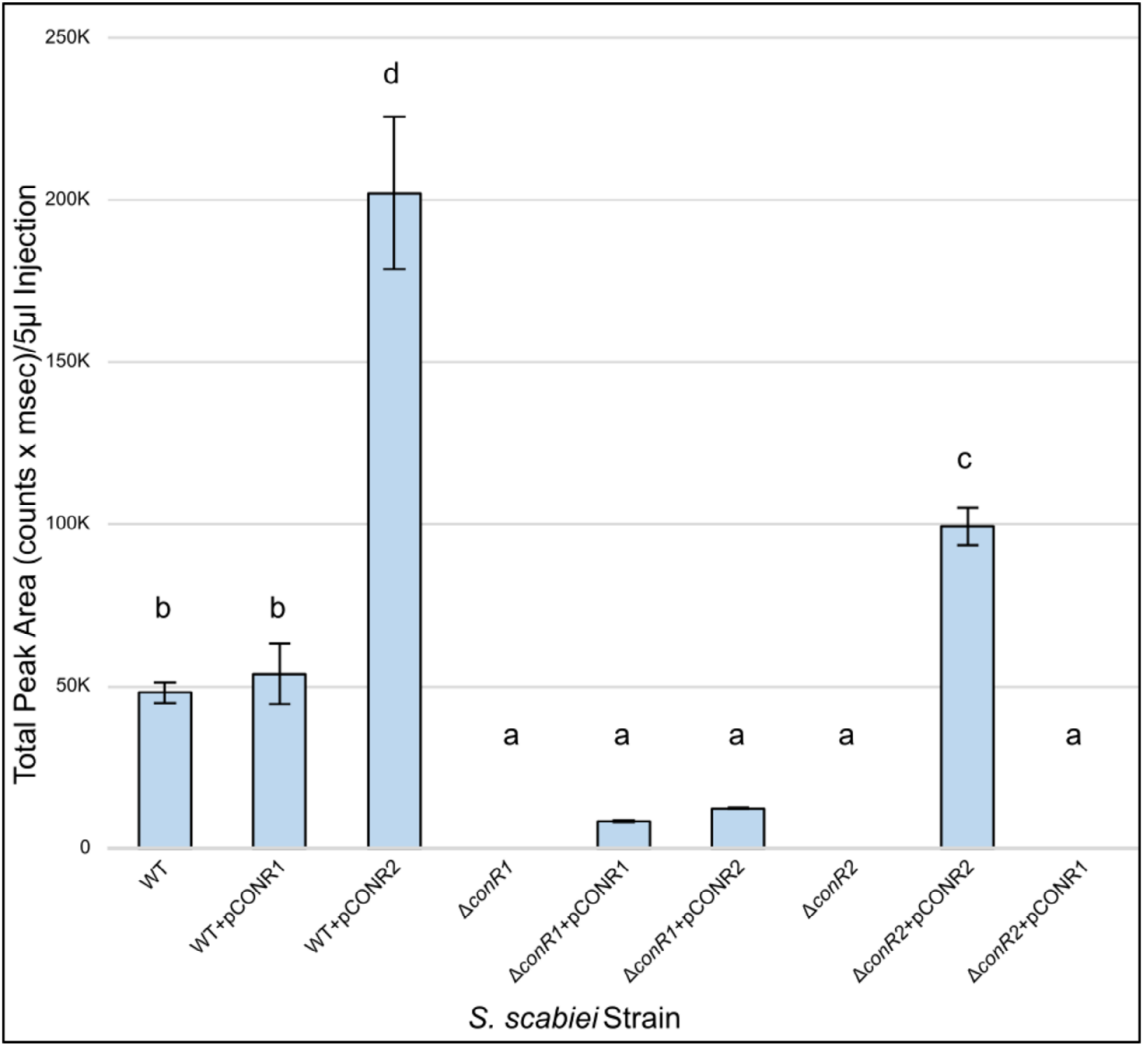
Production of total concanamycins on concanamycin production medium (CPM) agar by *Streptomyces scabiei* wild type (WT), gene deletion and gene overexpression strains. Each bar shows the mean metabolite production level (*n* = 3), and error bars represent the standard deviation from the mean. Means with different letters (a–d) were determined to be significantly different (*p* < 0.05). Vector control strains (WT + pRLDB50‐1a, Δ*conR1* + pRLDB50‐1a, Δ*conR2* + pRLDB50‐1a) were included in the analyses and did not show any differences from the WT, Δ*conR1*, and Δ*conR2* strains, respectively (data not shown).

To investigate the role of the two CSRs in concanamycin biosynthesis in *S. scabiei* , deletion mutants of each gene (Δ*conR1*, Δ*conR2*) were constructed (Figure S3). Culture extracts prepared from either mutant contained no detectable concanamycin metabolites (Figure 4; Figure S1), indicating that both CSRs are essential for metabolite biosynthesis. Similar findings were recently reported in the nonpathogenic species *Streptomyces eitanensis*, where CRISPRi‐mediated downregulation of the two CSRs in the concanamycin BGC reduced CMA levels to below detectable levels (Pereira et al. 2025). Concanamycin production in the Δ*conR1* and Δ*conR2* strains could be partially or fully rescued by heterologous expression of the corresponding gene (*conR1* or *conR2*) using the strong, constitutive *ermE*p* promoter (Figure 4). We also overexpressed each regulator in the WT strain, and although overexpression of *conR1* did not result in a significant increase in concanamycin production, overexpression of *conR2* did lead to significantly higher levels of concanamycin metabolites relative to the vector control (Figure 4). Additionally, cross‐complementation experiments revealed that although overexpression of *conR2* in the Δ*conR1* mutant could restore concanamycin production, overexpression of *conR1* in the Δ*conR2* mutant could not (Figure 4). This indicates that ConR2 is able to compensate for the loss of ConR1, but not vice versa. Overall, our results show that ConR1 and ConR2 are key positive activators of concanamycin biosynthesis in *S. scabiei*, and that ConR1 is likely higher in the regulatory cascade than ConR2 for controlling expression of the concanamycin biosynthetic genes. A previous study showed that expression of *conR2* is activated by the global regulators BldC, BldD, and BldG, which also control expression of the CSR for ThxA biosynthesis (Bignell et al. 2014). Whether these regulators additionally control expression of *conR1* is currently unknown and is the subject of future studies. The concanamycin BGC also contains six UUA codons, with four of these located within *conR1*, one in *conR2*, and one in the *conAII* PKS gene. UUA codons are rare in *Streptomyces* coding sequences, and expression of UUA‐containing genes is dependent on the *bldA* tRNA (Chater and Chandra 2008). It is therefore expected that expression of *conR1* and *conR2* and production of concanamycins are subjected to post‐transcriptional regulation by *bldA*, an idea that is currently under investigation.

### 2.4 Concanamycin Production Enhances the Virulence of *S. scabiei* on Radish Seedlings and Potato Tuber Tissue

**FIGURE 5:**
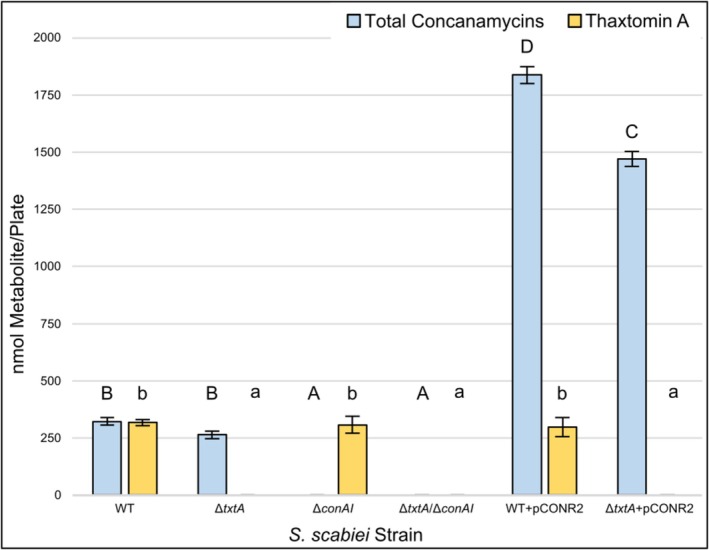
Production of total concanamycins and ThxA by *Streptomyces scabiei* wild type (WT), gene deletion and gene overexpression strains. For ThxA production, oat bran agar (OBA) was used for strain culturing, whereas concanamycin production medium (CPM) was used for assessing concanamycin production. Each bar shows the mean metabolite production level (*n* = 3), and error bars represent the standard deviation from the mean. Means with different letters (A–D for concanamycins; a, b for ThxA) were determined to be significantly different (*p* < 0.05).

Concanamycins can inhibit seedling growth of various plants (Natsume et al. 2005), and CMA has been shown to have a synergistic effect on the phytotoxicity of ThxA against excised potato tuber tissue (Natsume et al. 2017). Furthermore, CMA was detected along with ThxA in deep‐pitted CS scab lesions on potato tubers (Natsume et al. 2017). This suggests that concanamycin production may enhance the virulence of *S. scabiei* during plant–pathogen interactions. To explore this idea, we constructed a concanamycin‐deficient strain of *S. scabiei* 87‐22 by deleting the *conAI* gene, which encodes the first PKS in the concanamycin BGC (Figure 1). The gene was deleted in both the WT strain and a ThxA‐deficient mutant (Δ*txtA*) so that the impact of concanamycin production could be assessed both in the presence and in the absence of ThxA. HPLC analysis confirmed that concanamycin production was abolished in the constructed Δ*conAI* and Δ*txtA/*Δ*conAI* strains and that ThxA production was unaffected in the Δ*conAI* mutant (Figure 5; Figure S1). We also overexpressed *conR2* in both the WT and Δ*txtA* strains and showed that both strains produced high levels of concanamycins, whereas ThxA production levels were unaffected in the WT background (Figure 5). The vector control strains (WT + pRLDB50‐1a and Δ*txtA* + pRLDB50‐1a) did not show any difference from the WT and Δ*txtA* strains, respectively, with respect to relative production of concanamycins or ThxA (data not shown).

**FIGURE 6:**
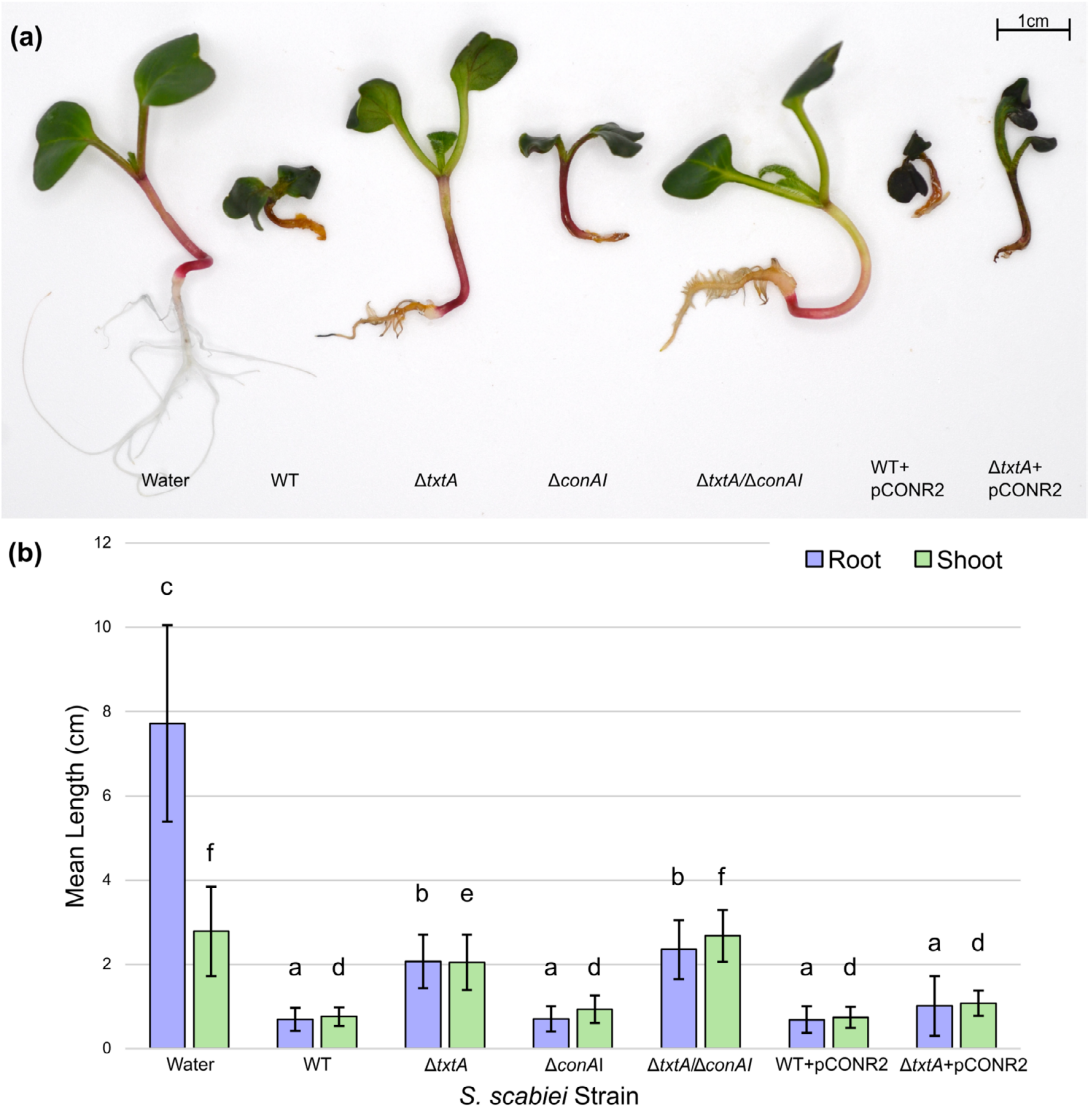
Virulence phenotype of *Streptomyces scabiei* strains (wild type [WT] and various mutants) on radish seedlings. Seeds treated with sterile distilled water served as a negative control. (a) Representative plants for each treatment are shown. (b) Quantification of the virulence phenotype. The average root and shoot measurements from 24 plants per treatment are indicated, with error bars representing standard deviations. Means with different letters (a–c for root length; d–f for shoot length) were determined to be significantly different (*p* < 0.05).

**FIGURE 7:**
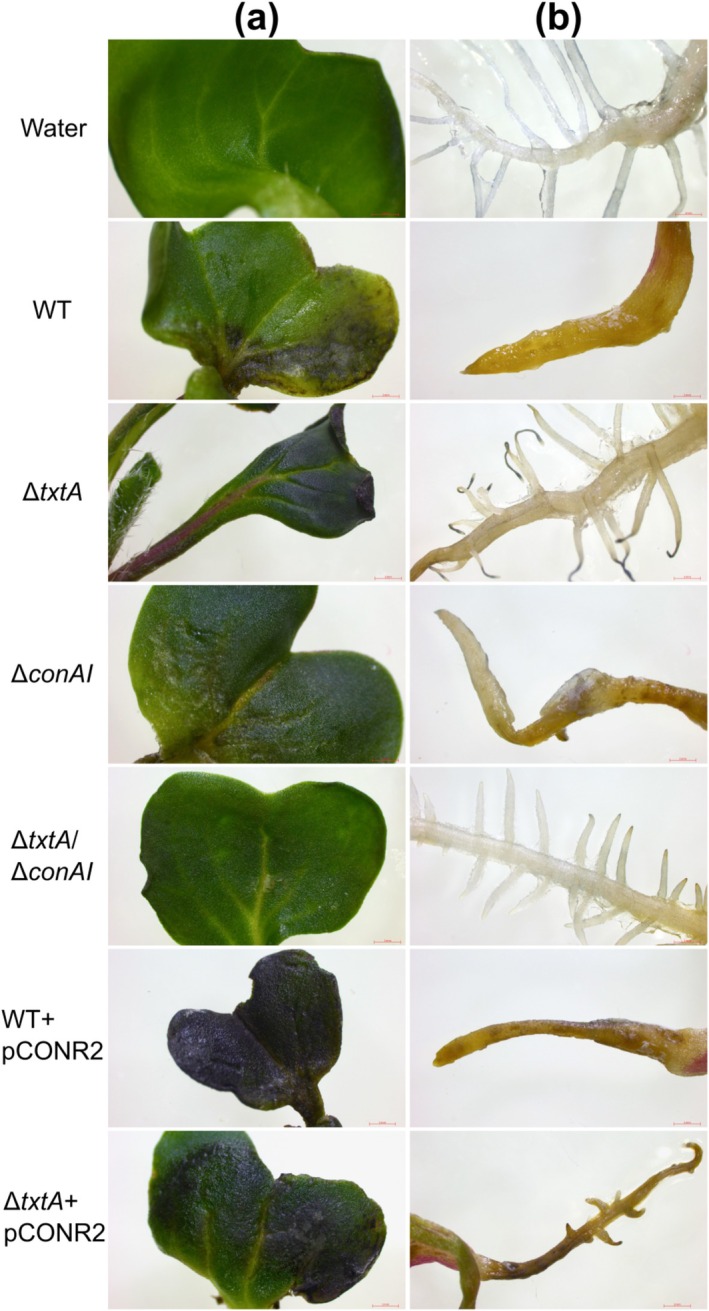
Morphological effects of *Streptomyces scabiei* infection on radish seedling (a) cotyledons and (b) tap roots. Treatment with sterile distilled water served as a negative control. WT, wild type. Representative cotyledons and roots (not necessarily from the same seedling) for each treatment are shown. All images are adjusted to the same scale. The scale bar in each photograph is equivalent to 1 mm.

Two different plant bioassays, a radish seedling and potato tuber slice bioassay, were conducted to assess the virulence phenotype of each strain. Seedlings infected with the Δ*conAI* strain showed similar stunting of roots and shoots as WT‐infected plants (Figure 6); however, the former displayed reduced visible necrosis of the cotyledons (Figure 7). When compared to the Δ*txtA‐*infected seedlings, the Δ*txtA/*Δ*conAI‐*infected seedlings showed significantly less stunting of the shoots (Figure 6), reduced necrosis of the cotyledons, and improved lateral root development (Figure 7). Elevated production of concanamycins by the WT + pCONR2 strain resulted in greater visible necrosis in the cotyledons of infected plants, whereas elevated concanamycin production in the absence of ThxA production by the Δ*txtA* + pCONR2 strain was associated with more severe visible necrosis in both the cotyledons and roots, and more severe stunting of the roots and shoots when compared to the Δ*txtA‐*infected plants (Figures 6 and 7). The vector control strains (WT + pRLDB50‐1a and Δ*txtA* + pRLDB50‐1a) were also included in the radish seedling bioassay and did not show any significant difference in virulence from the WT and Δ*txtA* strains, respectively (data not shown).

**FIGURE 8:**
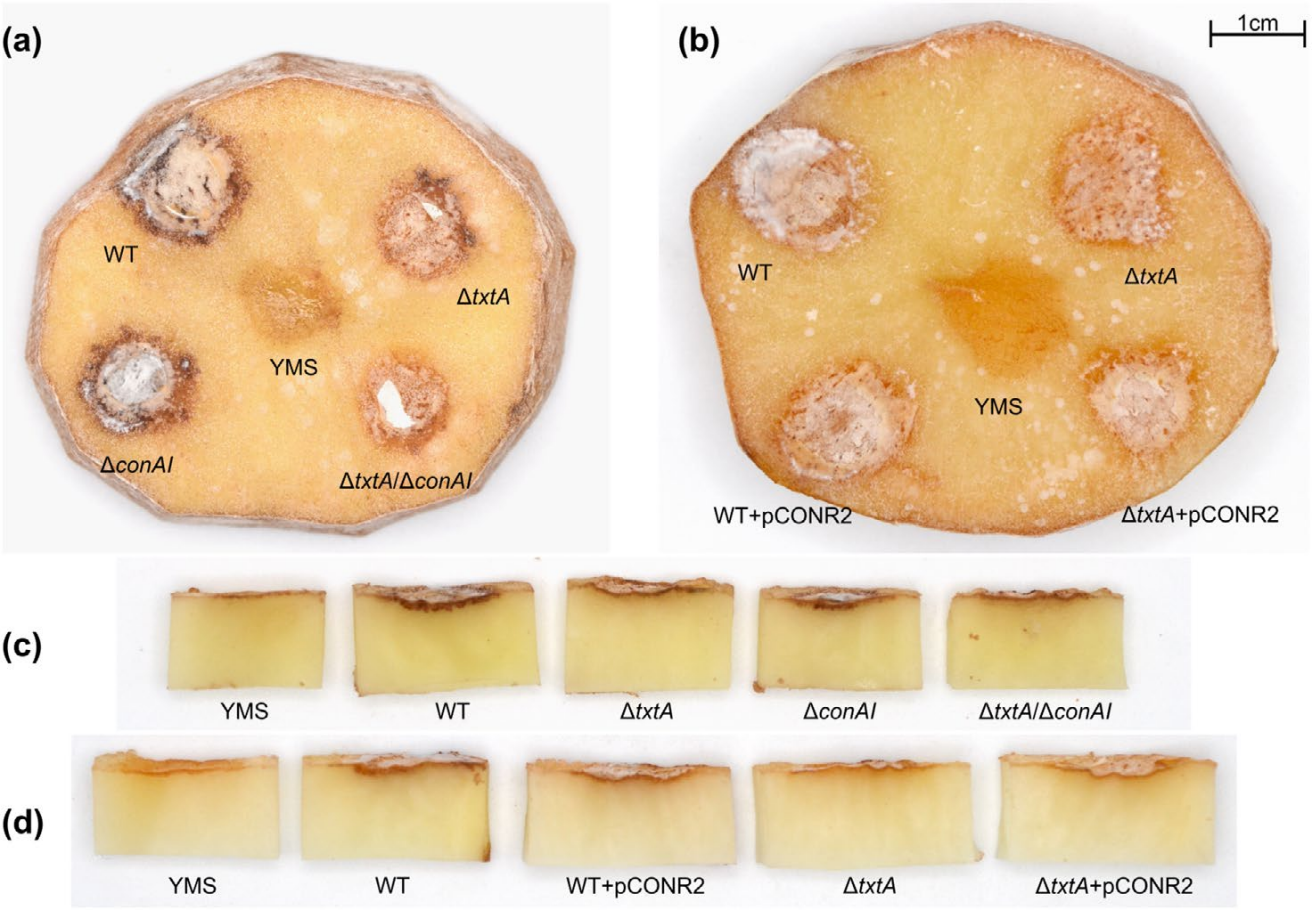
Virulence phenotype of *Streptomyces scabiei* strains on potato tuber slices. Uninoculated yeast extract‐malt extract‐starch (YMS) medium served as a negative control. Top view (a) and lateral view (c) of the slice inoculated with wild type (WT), Δ*txtA*, Δ*conAI*, and Δ*txtA/*Δ*conAI* strains. Top view (b) and lateral view (d) of the slice inoculated with WT, Δ*txtA*, WT + pCONR2, and Δ*txtA* + pCONR2 strains.

In the potato tuber slice bioassay, the Δ*conAI* mutant was typically associated with reduced pitting of the tuber tissue compared to the WT strain, whereas both strains caused comparable browning of the tissue at the inoculation site (Figure 8). The Δ*txtA* and Δ*txtA/*Δ*conAI* strains both caused reduced browning of the tuber tissue, which is attributed to the loss of ThxA production (Isayenka and Beaudoin 2022), and the Δ*txtA/*Δ*conAI* strain additionally caused little to no visible pitting (Figure 8). Elevated production of concanamycins by the WT + pCONR2 and the Δ*txtA* + pCONR2 strains was consistently associated with more severe pitting of the tuber tissue as compared to the WT and Δ*txtA* strains, respectively, whereas there was no visible increase in tissue browning (Figure 8). The vector control strains (WT + pRLDB50‐1a and Δ*txtA* + pRLDB50‐1a) displayed similar effects as the WT and Δ*txtA* strains (data not shown).

Overall, the bioassay results show that loss of concanamycin production reduces the virulence of *S. scabiei,* whereas elevated production enhances the virulence of this organism, and these effects are more pronounced in the absence of the ThxA phytotoxin. In a previous study published by Natsume et al. (2017), it was proposed that concanamycin production by *S. scabiei* may contribute to the formation of deep‐pitted scab lesions on potato tubers rather than the corky, raised lesions caused by *S. acidiscabies*, which does not produce concanamycins. Our results support this hypothesis as concanamycin production by *S. scabiei* was consistently associated with the pitting of potato tuber tissue in our bioassays.

It is noteworthy that the *S. scabiei* 87‐22 strain used in this study produces higher levels of ThxA compared to other *S. scabiei* strains (Loria et al. 1995) and that different strains of *S. scabiei* produce different relative amounts of ThxA and CMA/CMB (Natsume et al. 2001). ThxA is the primary pathogenicity determinant of *S. scabiei* and is essential for CS disease development, and it can be challenging to assess the importance of other virulence factors in the presence of high ThxA production levels. Nonetheless, our results support the notion that concanamycins function as important virulence factors in *S. scabiei* . Whether concanamycin production plays an additional role during plant host infection beyond symptom development is the subject of ongoing research in our laboratory.

## 3 Conclusions

Through mutational studies, we have provided direct evidence that concanamycins contribute to host–pathogen interactions by enhancing the severity of disease symptoms induced by *S. scabiei* during infection. Though the concanamycin BGC is not restricted to plant pathogens, it is highly conserved in several scab‐causing *Streptomyces* species, suggesting that these phytotoxins are relevant to the development of CS by multiple phytopathogenic *Streptomyces* spp. We also provided new insights into the genetic and nutritional factors that regulate the production of these compounds in *S. scabiei*, and we showed that the production of ThxA and concanamycins is affected by different nutritional signals. Notably, we found that although cello‐oligosaccharides can induce the production of ThxA, they do not appear to play a significant role in inducing concanamycin biosynthesis. This may indicate that ThxA and concanamycins play key roles at distinct stages of the infection process, an intriguing line of inquiry that deserves further investigation.

## 4 Experimental Procedures

### 4.1 Bacterial Strains, Culture Conditions, and Maintenance

Bacterial strains used in this study are listed in Table 1. *Escherichia coli* strains were routinely cultured at 37°C with shaking (200 rpm) in Difco LB Lennox broth (BD Biosciences), low‐salt LB broth (1% wt/vol tryptone, 0.5% wt/vol yeast extract, 0.25% wt/vol NaCl), Super Optimal Broth (SOB), or Super Optimal Broth with catabolite repression (SOC) (Hanahan 1983), or on LB (Lennox or low‐salt) medium with 1.5% wt/vol agar (BD Biosciences). When required, the media were supplemented with the following antibiotics at the indicated final concentrations: ampicillin (100 μg/mL), kanamycin (50 μg/mL), hygromycin B (100 μg/mL), chloramphenicol (25 μg/mL). For hygromycin B, low‐salt LB medium was used. *E. coli* strains were maintained at −80°C as cell suspensions in 20% vol/vol glycerol or at 4°C as plate cultures (Sambrook and Russell 2001).

**TABLE 1:** Bacterial strains, plasmids, and cosmids used in this study.

Strain, plasmid, or cosmid	Description	Resistance^a^	Reference or source
*Escherichia coli* strains			
DH5α	General cloning host	n/a	Gibco‐BRL
ET12567	*dam^−^, dcm^−^, hsdS^−^, hsdM^−^, hsdR^−^*; nonmethylating conjugation host	Cml^R^, Tet^R^, Strep^R^	MacNeil et al. (1992)
BW25113	Host for REDIRECT PCR targeting system; K‐12 derivative: Δ*araBAD*, Δ*rhaBAD*	n/a	Datsenko and Wanner (2000)
*Streptomyces scabiei* strains			
87‐22	Wild‐type strain	n/a	Loria et al. (1995)
Δ*txtA*	87‐22 derivative containing deletion of the *txtA* gene; thaxtomin‐deficient mutant	Apra^R^	Johnson et al. (2009)
Δ*conAI*	87‐22 derivative containing deletion of the *conAI* (*scab83871*) gene; concanamycin‐deficient mutant	Hyg^R^	This study
Δ*txtA/*Δ*conAI*	Δ*txtA* derivative containing deletion of the *conAI* gene, thaxtomin, and concanamycin deficient mutant	Apra^R^, Hyg^R^	This study
WT + pCONR2	87‐22 derivative harbouring the pCONR2 integrative plasmid; *conR2* overexpression/concanamycin overproduction mutant	Apra^R^, Thio^R^	This study
Δ*txtA +* pCONR2	Δ*txtA* derivative harbouring the pCONR2 integrative plasmid; thaxtomin‐deficient concanamycin‐overproduction mutant	Apra^R^, Thio^R^	This study
Δ*conR1*	87‐22 derivative containing deletion of the *conR1 (scab83841)* gene	Hyg^R^	This study
Δ*conR2*	87‐22 derivative containing deletion of the *conR2 (scab84101)* gene	Hyg^R^	This study
WT + pCONR1	87‐22 derivative harbouring the pCONR1 integrative plasmid; *conR1* overexpression mutant	Apra^R^, Thio^R^	This study
Δ*conR1* + pCONR1	Δ*conR1* strain complemented with pCONR1	Hyg^R^, Apra^R^, Thio^R^	This study
Δ*conR1* + pCONR2	Δ*conR1* strain cross‐complemented with pCONR2	Hyg^R^, Apra^R^, Thio^R^	This study
Δ*conR2* + pCONR2	Δ*conR2* strain complemented with pCONR2	Hyg^R^, Apra^R^, Thio^R^	This study
Δ*conR2* + pCONR1	Δ*conR2* strain cross‐complemented with pCONR1	Hyg^R^, Apra^R^, Thio^R^	This study
Plasmids or cosmids			
pUZ8002	Supplies transfer functions for mobilisation of *oriT*‐containing vectors from *E. coli* ET12567 to *Streptomyces*	Kan^R^	Paget et al. (1999)
pIJ790	pKD20 derivative; λ‐RED recombination plasmid (*gam, bet, exo*), *araC, rep101* ^ts^	Cml^R^	Gust, Challis, et al. (2003)
pIJ10700	Template for PCR amplification of the [*hyg* + *oriT*] cassette used for Redirect PCR targeting	Hyg^R^	Gust, O'Rourke, et al. (2003)
pGEM‐T Easy	General cloning vector	Amp^R^	Promega
pRLDB50‐1a	pLST9828 derivative; *Streptomyces* integrative expression vector; contains strong constitutive ermEp* promoter and integrates into C31 *attB* site	Apra^R^, Thio^R^	Bignell, Seipke, et al. (2010)
pCONR1	pRLDB50‐1a derivative containing *conR1* (*scab83841*) downstream of *ermE*p*; overexpresses *conR1* from the concanamycin biosynthetic gene cluster	Apra^R^, Thio^R^	This study
pCONR2	pRLDB50‐1a derivative containing *conR2* (*scab84101*) downstream of ermEp*; overexpresses *conR2* from the concanamycin biosynthetic gene cluster	Apra^R^, Thio^R^	This study
Cosmid 1732	Supercos1 derivative containing *conR1 (scab83841*) and *conAI* (*scab83871*) from *S. scabiei* 87‐22	Kan^R^, Amp^R^	This study
Cosmid1732/Δ*conR1*	Cosmid 1732 derivative, in which the *conR1* gene was replaced with the [*hyg* + *oriT*] cassette	Kan^R^, Amp^R^, Hyg^R^	This study
Cosmid1732/Δ*conAI*	Cosmid 1732 derivative, in which the *conAI* gene was replaced with the [*hyg* + *oriT*] cassette	Kan^R^, Amp^R^, Hyg^R^	This study
Cosmid 179	Supercos1 derivative containing *conR2* (*scab84101*) from *S. scabiei* 87‐22	Kan^R^, Amp^R^	This study
Cosmid179/Δ*conR2*	Cosmid 1732 derivative, in which the *conR2* gene was replaced with the [*hyg* + *oriT*] cassette	Kan^R^, Amp^R^, Hyg^R^	This study

^a^ Amp^R^, Apra^R^, Cml^R^ Hyg^R^, Kan^R^, Strep^R^, Tet^R^ and Thio^R^ = ampicillin, apramycin, chloramphenicol, hygromycin B, kanamycin, streptomycin, tetracycline and thiostrepton resistance, respectively.

*S. scabiei* strains were routinely cultured at 28°C. Liquid cultures using trypticase soy broth (TSB; BD Biosciences) were incubated with shaking (200 rpm) in 50 mL spring flasks. Solid cultures were grown at 28°C for 7–14 days on ISP‐4 (BD Biosciences), PMA (Fyans et al. 2015), soy flour mannitol agar (SFMA) (Kieser et al. 2000), Difco nutrient agar (NA; BD Biosciences), OBA (Johnson et al. 2007), maltose‐yeast extract‐malt extract agar (MYM) (Stuttard 1982), yeast extract‐malt extract‐starch agar (YMS) (Ikeda et al. 1987) and CPM (Haydock et al. 2005). When required, the media were supplemented with the following antibiotics at the indicated final concentrations: hygromycin B (100 μg/mL), apramycin (50 μg/mL), nalidixic acid (50 μg/mL), kanamycin (50 μg/mL), and thiostrepton (25 μg/mL). *S. scabiei* strains were maintained at −80°C as spore suspensions in 20% vol/vol glycerol or at 4°C as plate cultures (Kieser et al. 2000).

### 4.2 Plasmids, Cosmids, Primers, and DNA Manipulation

Plasmids and cosmids used in this study are listed in Table 1 and were manipulated using standard procedures (Sambrook and Russell 2001). Standard desalted oligonucleotide primers used for PCR or sequencing were purchased from Integrated DNA Technologies and are listed in Table S2. All DNA sequencing was performed at The Centre for Applied Genomics (Toronto, Canada).

### 4.3 Analysis of Thaxtomin A and Concanamycin Production

Seed cultures of *S. scabiei* strains were prepared by inoculating 50 μL of a dense spore stock into 10 mL of TSB and incubating for 72 h. Then, 100 μL of each seed culture was spread onto agar plates (15 × 60 mm Petri plates, 15 mL medium per plate) in triplicate. The media used were PMA, OBA, SFM, MYM, YMS, and CPM. The plates were incubated for 14 days, after which the cultures (mycelia and agar) were transferred to clean 50 mL conical tubes and were mashed thoroughly using a sterile metal spatula. The cultures were then stored at −80°C overnight. The tubes were thawed, ethyl acetate (10 mL) was added to each tube, and the contents were mixed and left overnight at room temperature in the dark. The organic extracts were transferred to clean conical tubes, and the remaining agar was rinsed once with another 5 mL of ethyl acetate. The extracts for each treatment were combined and dried using a dry nitrogen blower with heating to 50°C. The dried extracts were redissolved in 600 μL of HPLC‐grade methanol or ACN.

To assess concanamycin production on liquid and solid CPM, a seed culture of WT *S. scabiei* was prepared as described above. CPM agar plates (15 × 100 mm Petri plates, 30 mL medium per plate) were overlaid with sterile cellophane discs and were then each inoculated with 200 μL of seed culture. CPM broth cultures (3 × 125 mL spring flasks, 40 mL medium per flask) were each inoculated with 400 μL of seed culture. The plates and flasks were then incubated. At different time points (2, 4, 7, 10, and 14 days post‐inoculation), three plates were removed, and the mycelia were scraped off, dried, and then weighed to determine the dry cell weight (DCW). The remaining agar was transferred to 50 mL conical tubes and was processed, as described above for the extraction of metabolites. The resulting extracts were redissolved in 1 mL of HPLC‐grade ACN. For the liquid cultures, a 1 mL sample was removed at each time point, dried, and weighed to determine the DCW. A 2.5 mL sample was also removed from each culture and was stored at −80°C. The frozen samples were then thawed and extracted three each with 1 mL of ethyl acetate. The extracts for each treatment were combined and dried down using a dry nitrogen blower with heating to 50°C. The dried extracts were redissolved in 100 μL of HPLC‐grade ACN.

To assess concanamycin and ThxA production in media containing cellobiose and/or suberin, a seed culture of WT *S. scabiei* was prepared as described above. CPM agar plates (15 × 60 mm Petri plates, 15 mL medium per plate) were overlaid with sterile cellophane discs and were then inoculated with 100 μL of seed culture. The plates were incubated for 14 days, after which the mycelia were scraped off, dried, and then weighed to determine the DCW. The remaining agar was transferred to 50 mL conical tubes and was processed as described above for the extraction of metabolites. The dried extracts were redissolved in 600 μL of HPLC‐grade ACN.

Detection of ThxA and concanamycins was by HPLC using a 1260 Infinity Quaternary LC system (Agilent Technologies). Samples (5 μL) were loaded onto a Poroshell 120 EC‐C18 column (4.6 × 50 mm, 2.7 μm particle size) and eluted using an isocratic mobile phase consisting of either 70:30 ACN:water for concanamycins, or 30:70 ACN:water for ThxA, with a constant flow rate of 1 mL/min. The column temperature was held constant at 40°C. Concanamycins were monitored at 245 nm and ThxA was monitored at 380 nm using a diode array detector. Data acquisition and analysis was conducted using ChemStation software v. B.04.03 (Agilent Technologies Canada Inc.). Quantification of ThxA was achieved by generating a standard curve using known amounts of pure ThxA (Cayman Chemicals). To report total concanamycins, the peak areas for CMA, CMB, O‐methyl CMA, O‐methyl CMB, CMG and CMF were added together, and production levels were reported as either peak area (counts × msec) or as nmol total concanamycins. In the latter case, a standard curve generated using known amounts of pure CMA (Cayman Chemicals) was used to determine the ng of total concanamycin in each culture, and this was converted to nmol using the molecular weight of CMA (866.1 g/mol). When DCW measurements were obtained for a culture, the ThxA or concanamycin levels were reported as nmol (or peak area) per mg DCW.

### 4.4 LC–MS Analysis of Concanamycins

Liquid chromatography‐coupled mass spectrometry (LC–MS) analysis of culture extracts was conducted using a 1260 Infinity LC‐6230 TOF LC–MS system (Agilent). Extracts (10 μL) were loaded onto a ZORBAX SB‐C18 column (4.6 × 150 mm, 5 μm particle size) held at a constant temperature of 40°C. Separation was achieved using an isocratic mobile phase consisting of 70:30 ACN:water with a constant flow rate of 1 mL/min. Mass spectra were recorded in positive mode between 200 and 2500 *m/z*. Data acquisition and analysis were performed using MassHunter B.08.00 (Agilent).

### 4.5 Targeted Deletion of *S. scabiei* 87–22 Genes

Deletion of *conAI* (*scab83871*), *conR1* (*scab83841*), and *conR2 (scab84101*) was accomplished using the previously described REDIRECT PCR targeting system (Gust, Challis, et al. 2003; Gust, O'Rourke, et al. 2003). A cassette (*hyg‐oriT*) containing the hygromycin resistance gene and origin of transfer sequence was PCR amplified using pIJ10700 as the template and primers CV1/CV2, CV5/CV6, CV9/CV10 for targeting of the *conAI, conR1*, and *conR2* genes, respectively (Table S2). Following gel purification, the PCR product was electroporated into *E. coli* BW25113/pIJ790 containing Cosmid 1732 (for *conAI* and *conR1*) or Cosmid 179 (for *conR2*) (Table 1). Mutant cosmids were isolated and verified by PCR using the primers HygF/HygR and flanking primers for each target gene (Table S2). A single mutant cosmid was subsequently introduced into *E. coli* ET12567/pUZ8002 before being transferred to wild‐type *S. scabiei* 87–22 via intergeneric conjugation as described before (Kieser et al. 2000). The Δ*conAI* mutant cosmid was also transferred into the thaxtomin‐deficient mutant Δ*txtA*. Hygromycin‐resistant exconjugants were screened for kanamycin sensitivity, and the constructed mutant strains (three isolates per gene deletion) were verified by PCR (Figure S3).

### 4.6 Overexpression of *conR1* and *conR2* in *S. scabiei*

The *conR1* and *conR2* gene sequences were amplified by PCR using Cosmid 1732 and Cosmid 179 as templates, respectively, and the primers CV19/CV20 and CV21/CV22, respectively (Table S2). The resulting products were cloned into pGEM T‐Easy (Table 1), sequenced, then released by digestion of the pGEM clones with XbaI to give a 3011 bp fragment for *conR1*, or with BamHI to give a 2300 bp fragment for *conR2*. After gel purification, the *conR1‐* and *conR2‐*containing DNA fragments were individually ligated into XbaI‐ or BamHI‐digested pRLDB50‐1a to give pCONR1 and pCONR2, respectively. The presence and correct orientation of the insert were confirmed by PCR with primers ermEp*for1/CV23 for pCONR1 and ermEp*for1/CV24 for pCONR2. Each of pRLDB50‐1a, pCONR1, and pCONR2 was then introduced into *S. scabiei* WT, Δ*conR1*, and Δ*conR2* via intergeneric conjugation with *E. coli*. Both pRLDB50‐1a and pCONR2 were also introduced into the thaxtomin‐deficient mutant, Δ*txtA*, via intergeneric conjugation with *E. coli*. PCR with primers pSETF/pSETR was used to confirm the successful integration of the vector in the apramycin/thiostrepton‐resistant exconjugants that arose. For each constructed strain, two isolates were selected and verified.

### 4.7 Virulence Bioassays

To assess the virulence phenotype of the *S. scabiei* strains, an in vitro radish seedling assay was performed. Radish seeds (cv. Cherry Belle; McKenzie Seeds) were surface sterilised by treating with 70% vol/vol ethanol for 5 min followed by treatment with 17% vol/vol bleach (Clorox) and 0.1% vol/vol polysorbate (Tween) 20 for 10 min with gentle agitation. The seeds were washed ≥ 5 times with sterile distilled water and then placed into a sterile Petri dish containing Whatman No. 1 filter paper moistened with sterile water and nystatin (50 μg/mL final concentration), wrapped with Parafilm, and then left in the dark at room temperature for 48 h to germinate. Seed cultures of *S. scabiei* strains were prepared by inoculating 50 μL of a dense spore stock into 10 mL of TSB and incubating for 72 h. The mycelia were then pelleted, washed twice with sterile water, weighed, and resuspended in 3 volumes of water (e.g., 0.3 g mycelia: 0.9 mL water). Phytagel (0.6% wt/vol) in 0.5× Murashige and Skoog (MS) medium (Phytotech Labs) (pH 6.8) was poured into deep Petri dishes (100 mm × 25 mm; 50 mL per dish). Wells (six per dish) were cut in the agar using the end of a sterile 13 mm diameter test tube, and the bottom of each well was sealed with 200 μL of molten MS agar. One germinated seed was placed into each well, and each seed was treated with 100 μL of mycelial suspension or sterile distilled water as control. A total of two plates (12 seeds) per treatment were set up. The plates were wrapped with Parafilm and incubated at 21°C under a 16‐h photoperiod for 7 days. The seedlings were then photographed, and measurements of root (tap root) and shoot (hypocotyl) lengths were conducted using ImageJ (Schneider et al. 2012). Outliers, namely, the two largest and two smallest seedlings, were removed each time, leaving data for eight seedlings per treatment. The assay was performed three times in total, and data from the three repeats were combined for a total of 24 seedlings per treatment.

A potato tuber slice assay was performed as described previously (Loria et al. 1995), with some modifications. Seed cultures of *S. scabiei* strains were prepared as described above. A 200 μL aliquot of each culture was then spread‐plated onto YMS agar, and the plates were incubated at 28°C until well sporulated (10 days). Potatoes from the grocery store (Canada No. 1 White Potatoes) were peeled, disinfected in 15% vol/vol bleach, rinsed twice with sterile water, and cut into ~2 cm thick slices. The slices were placed into sterile glass Petri dishes containing filter paper pre‐wetted with sterile water. Soft NA (50 μL, containing 0.8% wt/vol agar) was spotted on top of the slices, and agar cores (8 mm diameter) were cut from the *S. scabiei* YMS plates and placed spore‐side down on top of the soft NA spots. Cores from uninoculated YMS served as the negative control. The plates were wrapped with Parafilm and incubated in the dark at room temperature for 12 days, after which the potato slices were photographed. The assay was performed three times, with at least three replicates per strain each time.

### 4.8 Statistical Analysis

Calculations of means and standard deviations for metabolite analysis and radish seedling bioassay results, and visualisation of results, were performed in Microsoft Excel. The results of the radish seedling bioassay and metabolite analyses were analysed using a one‐way analysis of variance (ANOVA) with a post hoc Tukey test in either IBM SPSS Statistics or Minitab Statistical Software.

### 4.9 Bioinformatics Analysis

The *S. scabiei* 87‐22 genome (FN554889.1) has been previously sequenced and the concanamycin BGC identified (Bignell, Huguet‐Tapia, et al. 2010; Liu et al. 2021). Cblaster (Gilchrist et al. 2021) was used with genes from the *S. scabiei* 87‐22 BGC as a query to identify other *Streptomyces* genomes containing potential concanamycin BGCs (Table S3). The eight query genes used were the six PKS genes *conAI–conAVI* (*scab83871, scab83891, scab83901, scab83911, scab83921, scab83931*) and the two CSRs *conR1* (*scab83841*) and *conR2* (*scab84101*); the minimum number of unique query sequence hits was set to six. The hits were then run through antiSMASH v. 7.0 (Blin et al. 2023), and the antiSMASH‐generated region files were used to run the BiG‐SCAPE tool with the default parameters (Navarro‐Muñoz et al. 2020). The *S. neyagawaensis* concanamycin BGC (BGC0000040) from MIBiG (minimum information about a biosynthetic gene cluster) (Terlouw et al. 2023) was included as a reference. Network files were visualised using Cytoscape v. 3.9.0 (Shannon et al. 2003). The BGCs were also compared using Clinker with default parameters and Clustermap.js for BGC alignment visualisation (Gilchrist and Chooi 2021).

## Author Contributions

**Corrie V. Vincent:** conceptualization (supporting); methodology (supporting); investigation (lead); formal analysis (lead); visualisation (lead); writing – original draft (lead); and writing – review and editing (equal). **Dawn R. D. Bignell:** conceptualization (lead); funding acquisition (lead); supervision (lead); methodology (lead); and writing – review and editing (equal).

## Conflicts of Interest

The authors declare no conflicts of interest.

## Supporting information


**Figure S1:** Detection of concanamycins by high‐performance liquid chromatography. Shown are the chromatograms for the standards (i) CMA (1000 ng) and (ii) CMB (1000 ng) dissolved in ACN, and for the CPM agar culture extracts of (iii, iv) *S. scabiei* WT, (v) Δ*conAI*, (vi) Δ*conR1* and (vii) Δ*conR2*. The WT culture extract shown in (iii) was redissolved in MeOH, whereas the WT and mutant culture extracts shown in (iv, v, vi, vii) were redissolved in ACN. Peaks for CMA (1) and CMB (2) and additional concanamycin derivatives detected by HPLC–MS‐TOF are numbered as in Table S1.

**Figure S2:** Morphological development and production of total concanamycins by WT *S. scabiei* cultured on CPM. (a) WT *S. scabiei* growth on CPM agar over 14 days. (b) Total concanamycin production levels following growth on CPM agar and broth over 14 days. The resulting concanamycin peak areas were normalised using the corresponding dry cell weight (DCW) measurements. Each point shows the mean normalised metabolite production level (*n* = 3), and error bars represent the standard deviation from the mean. Representative results from duplicate experiments are shown.

**Figure S3:** PCR verification of Δ*conAI*, Δ*txtA/*Δ*conAI*, Δ*conR1*, and Δ*conR2* gene deletion mutants. (a) Schematic diagram showing the annealing sites of primers CV25/CV26 used for PCR verification of *conAI (scab83871)* deletion and expected product sizes in bp. (b) Agarose gel electrophoresis of the PCR products generated using primers CV25/CV26 with genomic DNA from *S. scabiei* WT (8.6 kb not amplified; lane 2), Δ*txtA* (8.6 kb not amplified; lane 3), Δ*conAI* × 3 isolates (lanes 4–6), Δ*txtA/ΔconAI* × 3 isolates (lanes 7–9), negative control with water in place of template DNA (lane 10). Band sizes were estimated by comparison with the 1 kb ladder (FroggaBio) in lanes 1&11. (c) Schematic diagram showing the annealing sites of primers CV15/CV16 for PCR verification of *conR1 (scab83841)* deletion and primers CV17/CV18 for PCR verification of *conR2 (scab84101)* deletion. Expected product sizes indicated in bp. (d) Agarose gel electrophoresis of the PCR products generated using primers CV15/CV16 with genomic DNA from *S. scabiei* WT (lane 2), Δ*conR1* × 3 isolates (lanes 3–5), negative control with water in place of template DNA (lane 6); primers CV17/CV18 with genomic DNA from *S. scabiei* 87–22 (lane 7), Δ*conR2* × 3 isolates (lanes 8–10), negative control with water in place of template DNA (lane 11). Size was estimated by comparison with the 1 kb ladder (FroggaBio) in lanes 1 and 12.

**Table S1:** List of concanamycins detected in *S. scabiei* culture extracts by HPLC–MS‐TOF analysis.

**Table S2:** Oligonucleotide primers used in this study.

**Table S3:** NCBI accessions used for antiSMASH analysis.

## Data Availability

The data that support the findings of this study are available from the corresponding author upon reasonable request.
