# Supplementary material for: Concanamycins Are Key Contributors to the Virulence of the Potato Common Scab Pathogen Streptomyces scabiei
Source: Mol Plant Pathol. 2025 Nov 26;26(11):e70175. doi: 10.1111/mpp.70175 (PMC12648119; doi:10.1111/mpp.70175)
Supplement: Supplementary file 1 — Figure S1: Detection of concanamycins by high‐performance liquid chromatography. Shown are the chromatograms for the standards (i) CMA (1000 ng) and (ii) CMB (1000 ng) dissolved in ACN, and for the CPM agar culture extracts of (iii, iv) S. scabiei WT, (v) ΔconAI, (vi) ΔconR1 and (vii) ΔconR2. The WT culture extract shown in (iii) was redissolved in MeOH, whereas the WT and mutant culture extracts shown in (iv, v, vi, vii) were redissolved in ACN. Peaks for CMA (1) and CMB (2) and additional concanamycin derivatives detected by HPLC–MS‐TOF are numbered as in Table S1. [file MPP-26-e70175-s003.docx]

**Figure S1.** Detection of concanamycins by high-performance liquid chromatography. Shown are the chromatograms for the standards **(i)** CMA (1000 ng) and **(ii)** CMB (1000 ng) dissolved in ACN, and for the CPM agar culture extracts of **(iii, iv)** *S. scabiei* WT, **(v)** Δ*conAI,* **(vi)** Δ*conR1* and **(vii)** Δ*conR2.* The WT culture extract shown in **(iii)** was redissolved in MeOH, while the WT and mutant culture extracts shown in **(iv, v, vi, vii)** were redissolved in ACN. Peaks for CMA (1) and CMB (2) and additional concanamycin derivatives detected by HPLC-MS-TOF are numbered as in Table S1.
