# Supplementary material for: Concanamycins Are Key Contributors to the Virulence of the Potato Common Scab Pathogen Streptomyces scabiei
Source: Mol Plant Pathol. 2025 Nov 26;26(11):e70175. doi: 10.1111/mpp.70175 (PMC12648119; doi:10.1111/mpp.70175)
Supplement: Supplementary file 2 — Figure S2: Morphological development and production of total concanamycins by WT S. scabiei cultured on CPM. (a) WT S. scabiei growth on CPM agar over 14 days. (b) Total concanamycin production levels following growth on CPM agar and broth over 14 days. The resulting concanamycin peak areas were normalised using the corresponding dry cell weight (DCW) measurements. Each point shows the mean normalised metabolite production level (n = 3), and error bars represent the standard deviation from the mean. Representative results from duplicate experiments are shown. [file MPP-26-e70175-s001.docx]

**
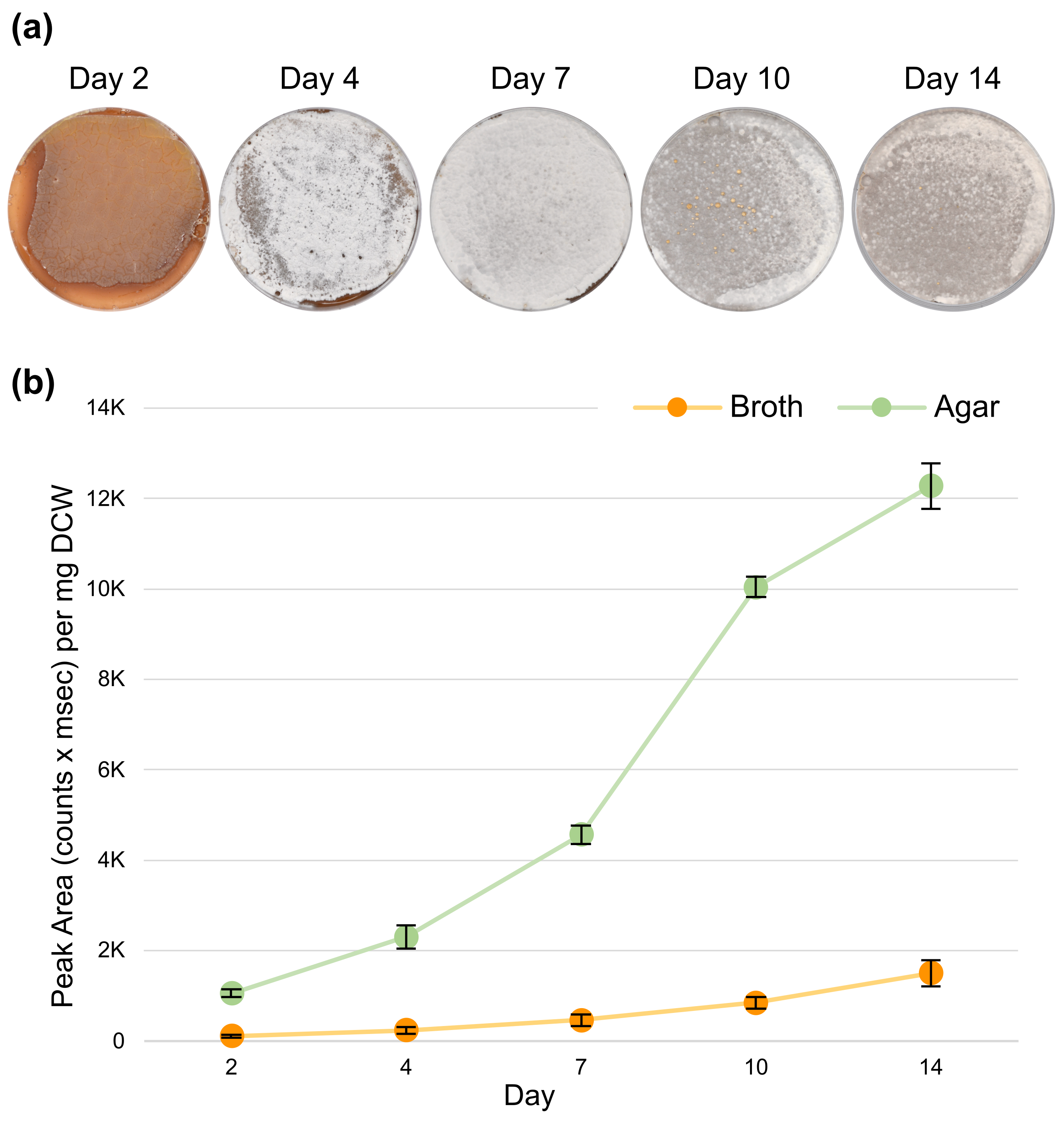
**

**Figure S2.** Morphological development and production of total concanamycins by WT *S. scabiei* cultured on CPM. (a) WT *S. scabiei* growth on CPM agar over 14 days. (b) Total concanamycin production levels following growth on CPM agar and broth over 14 days. The resulting concanamycin peak areas were normalized using the corresponding dry cell weight (DCW) measurements. Each point shows the mean normalized metabolite production level (n=3) and error bars represent the standard deviation from the mean. Representative results from duplicate experiments are shown.
