# Supplementary material for: Concanamycins Are Key Contributors to the Virulence of the Potato Common Scab Pathogen Streptomyces scabiei
Source: Mol Plant Pathol. 2025 Nov 26;26(11):e70175. doi: 10.1111/mpp.70175 (PMC12648119; doi:10.1111/mpp.70175)
Supplement: Supplementary file 3 — Figure S3: PCR verification of ΔconAI, ΔtxtA/ΔconAI, ΔconR1, and ΔconR2 gene deletion mutants. (a) Schematic diagram showing the annealing sites of primers CV25/CV26 used for PCR verification of conAI (scab83871) deletion and expected product sizes in bp. (b) Agarose gel electrophoresis of the PCR products generated using primers CV25/CV26 with genomic DNA from S. scabiei WT (8.6 kb not amplified; lane 2), ΔtxtA (8.6 kb not amplified; lane 3), ΔconAI × 3 isolates (lanes 4–6), ΔtxtA/ΔconAI × 3 isolates (lanes 7–9), negative control with water in place of template DNA (lane 10). Band sizes were estimated by comparison with the 1 kb ladder (FroggaBio) in lanes 1&11. (c) Schematic diagram showing the annealing sites of primers CV15/CV16 for PCR verification of conR1 (scab83841) deletion and primers CV17/CV18 for PCR verification of conR2 (scab84101) deletion. Expected product sizes indicated in bp. (d) Agarose gel electrophoresis of the PCR products generated using primers CV15/CV16 with genomic DNA from S. scabiei WT (lane 2), ΔconR1 × 3 isolates (lanes 3–5), negative control with water in place of template DNA (lane 6); primers CV17/CV18 with genomic DNA from S. scabiei 87–22 (lane 7), ΔconR2 × 3 isolates (lanes 8–10), negative control with water in place of template DNA (lane 11). Size was estimated by comparison with the 1 kb ladder (FroggaBio) in lanes 1 and 12. [file MPP-26-e70175-s004.docx]

**
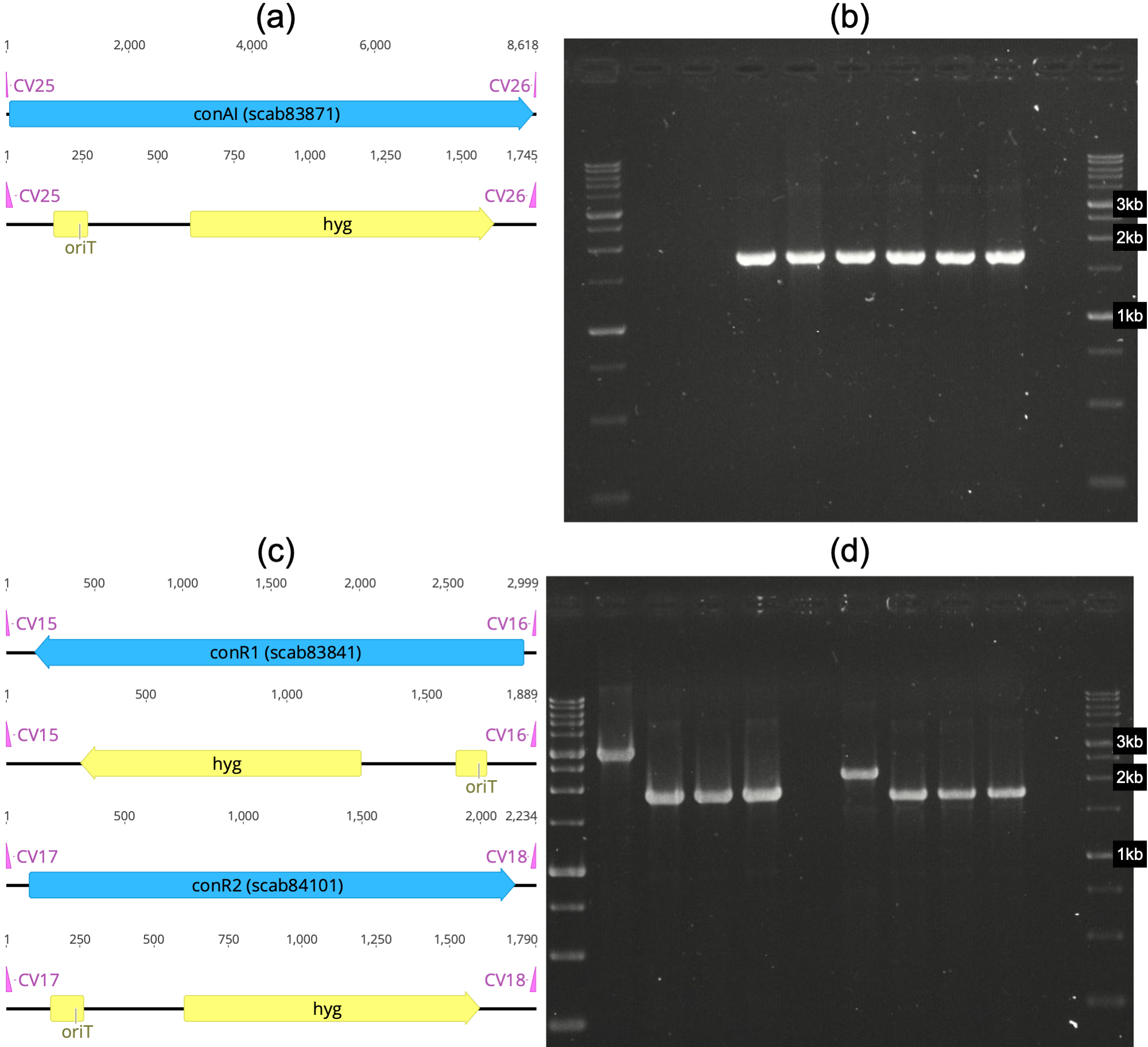
**

**Figure S3.** PCR verification of Δ*conAI,* Δ*txtA/*Δ*conAI,* Δ*conR1* and Δ*conR2* gene deletion mutants. (a) Schematic diagram showing the annealing sites of primers CV25/CV26 used for PCR verification of *conAI (scab83871)* deletion and expected product sizes in bp. (b) Agarose gel electrophoresis of the PCR products generated using primers CV25/CV26 with genomic DNA from *S. scabiei* WT (8.6kb not amplified; lane 2), Δ*txtA* (8.6kb not amplified; lane 3), Δ*conAI* × 3 isolates (lanes 4-6), Δ*txtA/ΔconAI* × 3 isolates (lanes 7-9), negative control with water in place of template DNA (lane 10). Band sizes were estimated by comparison with the 1kb ladder (FroggaBio) in lanes 1&11. (c) Schematic diagram showing the annealing sites of primers CV15/CV16 for PCR verification of *conR1 (scab83841)* deletion and primers CV17/CV18 for PCR verification of *conR2 (scab84101)* deletion. Expected product sizes indicated in bp. (d) Agarose gel electrophoresis of the PCR products generated using primers CV15/CV16 with genomic DNA from *S. scabiei* WT (lane 2), Δ*conR1* × 3 isolates (lanes 3-5), negative control with water in place of template DNA (lane 6); primers CV17/CV18 with genomic DNA from *S. scabiei* 87-22 (lane 7), Δ*conR2* × 3 isolates (lanes 8-10), negative control with water in place of template DNA (lane 11). Size was estimated by comparison with the 1kb ladder (FroggaBio) in lanes 1 and 12.
