# Supplementary material for: Concanamycins Are Key Contributors to the Virulence of the Potato Common Scab Pathogen Streptomyces scabiei
Source: Mol Plant Pathol. 2025 Nov 26;26(11):e70175. doi: 10.1111/mpp.70175 (PMC12648119; doi:10.1111/mpp.70175)
Supplement: Supplementary file 4 — Table S1: List of concanamycins detected in S. scabiei culture extracts by HPLC–MS‐TOF analysis. [file MPP-26-e70175-s005.docx]

**Table S1.** List of concanamycins detected in *Streptomyces scabiei* culture extracts by HPLC-MS-TOF analysis.

| **Number** | **Compound** | **Observed *m/z***  **(M+Na)^+^** | **Expected *m/z***  **(M+Na)^+^** | **Reference** |
| --- | --- | --- | --- | --- |
| 1 | Concanamycin A | 888.5080 | 888 | Woo et al. 1992 |
| 2 | Concanamycin B | 874.4923 | 874 | Kinashi et al. 1984 |
| 3 | Concanamycin F | 715.4392 | 715 | Woo et al. 1992 |
| 4 | Concanamycin G | 683.4130 | 683 | Woo et al. 1992 |
| 5 | Concanamycin H* | 697.4279 | 697.4279 | Li et al. 2022 |
| 6 | 21-O-Methyl Concanamycin A | 902.5236 | 903 | Ingenhorst et al. 2001 |
| 7 | 21-O-Methyl Concanamycin B** | 888.5080 | 888.5080 | Predicted** |

*Does not separate as a distinct peak from concanamycin A under conditions used, but *m/z* detected within the concanamycin A peak.

** *m/z* predicted based on molecular formula in Agilent MassHunter B.08.00 software (Agilent Technologies Canada Inc). Distinguished from concanamycin A by use of an authentic standard for concanamycin A and distinct retention times.

**References**

Ingenhorst, G., Bindseil, K.U., Boddien, C., Dröse, S., Gaßel, M., Altendorf, K., et al. (2001) Synthesis of a doubly labelled concanamycin derivative for ATPase binding studies. *European Journal of Organic Chemistry* 2001: 4525–4532. <https://doi.org/10.1002/1099-0690(200112)2001:23%3C4525::AID-EJOC4525%3E3.0.CO;2-S>.

Kinashi, H., Someno, K. & Sakaguchi, K. (1984) Isolation and characterization of concanamycins A, B and C. *The Journal of Antibiotics* 37: 1333–1343. <https://doi.org/10.7164/antibiotics.37.1333>.

Li, G.L., Qi, H.M., He, Y.L., Shen, Y.K. & Shen, T. (2022) Concanamycin H from the soil actinomycete *Streptomyces* sp. R1706-8. *Journal of Chemical Research* 46: 1–6. <https://doi.org/10.1177/17475198221109161>.

Woo, J.T., Shinohara, C., Sakai, K., Hasumi, K. & Endo, A. (1992) Isolation, characterization and biological activities of concanamycins as inhibitors of lysosomal acidification. *The Journal of Antibiotics* 45: 1108–1116. <https://doi.org/10.7164/antibiotics.45.1108>.
