# Supplementary material for: Concanamycins Are Key Contributors to the Virulence of the Potato Common Scab Pathogen Streptomyces scabiei
Source: Mol Plant Pathol. 2025 Nov 26;26(11):e70175. doi: 10.1111/mpp.70175 (PMC12648119; doi:10.1111/mpp.70175)
Supplement: Supplementary file 5 — Table S2: Oligonucleotide primers used in this study. [file MPP-26-e70175-s006.docx]

**Table S2.** Oligonucleotide primers used in this study

| **Primer** | **Sequence (5' → 3')*** | **Use** |
| --- | --- | --- |
| CV1 | ATGCCGATTGCGGTCGTCGGATACTCCTGCCGG CTGCCCATTCCGGGGATCCGTCGACC | Forward primer, *conAI* (*scab83871*) REDIRECT disruption cassette |
| CV2 | TCAGGACTCGATGCCTTCGCGGGCCATCCGGA CCAGTTCTGTAGGCTGGAGCTGCTTC | Reverse primer, *conAI* (*scab83871*) REDIRECT disruption cassette |
| CV25 | AAGCCGGAAATGGAAGAAATGC | Forward primer, detection of *conAI* gene or *hyg* disruption cassette (flanking primer, upstream of CV1); Δ*conAI* mutant verification |
| CV26 | CGTTCGGGTCATGAGCGTTC | Forward primer, detection of *conAI* gene or *hyg* disruption cassette (flanking primer, downstream of CV2); Δ*conAI* mutant verification |
| CV5 | TCAGATGGATCCGACGGCCAGGGCTTTGAACTCTCTGGGTGTAGGCTGGAGCTGCTTC | Forward primer, *conR1* (*scab83841*) REDIRECT disruption cassette |
| CV6 | ATGATACTGATCGACCGCAAAGACGAACTGTCCGCATTAATTCCGGGGATCCGTCGACC | Reverse primer, *conR1* (*scab83841*) REDIRECT disruption cassette |
| CV15 | GCTCGACGCCTTGTGTTG | Forward primer, detection of *conR1* gene or *hyg* disruption cassette (flanking primer, upstream of CV5); Δ*conR1* mutant verification |
| CV16 | AGGCCTCCGGCAATCTAC | Forward primer, detection of *conR1* gene or *hyg* disruption cassette (flanking primer, downstream of CV6); Δ*conR1* mutant verification |
| CV9 | ATGGAGTTCTTGTTGCTAGGTCCCCTGGTCATCCGGTCGATTCCGGGGATCCGTCGACC | Forward primer, *conR2* (*scab84101*) REDIRECT disruption cassette |
| CV10 | TCACCCGGACGCGGCCCGGGGCGCCACGACCGGTGGCAGTGTAGGCTGGAGCTGCTTC | Reverse primer, *conR2* (*scab84101*) REDIRECT disruption cassette |
| CV17 | CGACGGAAATCGCATCATG | Forward primer, detection of *conR2* gene or *hyg* disruption cassette (flanking primer, upstream of CV9); Δ*conR2* mutant verification |
| CV18 | GTGGGAGAAGGACGTCGAG | Forward primer, detection of *conR2* gene or *hyg* disruption cassette (flanking primer, downstream of CV10); Δ*conR2* mutant verification |
| HygF | CGCATAGACGTCGGTGAAGT | Forward primer, detection of *hyg* resistance gene; Δ*conAI,* Δ*conR1,* Δ*conR2* mutant verification |
| HygR | TACCTGGTGATGAGCCGGAT | Reverse primer, detection of *hyg* resistance gene; Δ*conAI,* Δ*conR1,* Δ*conR2,* mutant verification |
| CV19 | ATAT**TCTAGA**CTGCCGCTCGACGCCTTGTG | Forward primer, *conR1* (*scab83841*) coding sequence; XbaI site added |
| CV20 | ATAT**TCTAGA**GAGGCCTCCGGCAATCTACTCG | Reverse primer, *conR1* coding sequence; XbaI site added |
| CV21 | ATAT**GGATCC**CACTGTCGCGTCGCGTGGTG | Forward primer, *conR2* (*scab84101*) coding sequence; BamHI site added |
| CV22 | ATAT**GGATCC**GGGAGGTCGTGGGAGAAGGACG | Reverse primer, *conR2* coding sequence; BamHI site added |
| ermEp*for1 | GACCGGAGTTCGAGGTACG | Forward primer, verification of *conR1* and *conR2* inserts in pRLDB50-1a |
| CV23 | CCTCCTCGGCAAAGGCATAG | Reverse primer, verification of *conR1* insert in pRLDB50-1a |
| CV24 | AAGCTCGCGATGTTGTCCAC | Reverse primer, verification of *conR2* insert in pRLDB50-1a |
| pSETF | GAGCGGATAACAATTTCACACAGGA | Forward primer, verification of pRLDB50-1a, pCONR1, pCONR2 integration |
| pSETR | CGCCAGGGTTTTCCCAGTCA | Reverse primer, verification of pRLDB50-1a, pCONR1, pCONR2 integration |

*Non-homologous extensions are underlined, while engineered restriction endonuclease sites are indicated in bold
