# Supplementary material for: Concanamycins Are Key Contributors to the Virulence of the Potato Common Scab Pathogen Streptomyces scabiei
Source: Mol Plant Pathol. 2025 Nov 26;26(11):e70175. doi: 10.1111/mpp.70175 (PMC12648119; doi:10.1111/mpp.70175)
Supplement: Supplementary file 6 — Table S3: NCBI accessions used for antiSMASH analysis. [file MPP-26-e70175-s002.docx]

**Table S3.** NCBI accessions used for antiSMASH analysis

| **Species name** | **Accession** |
| --- | --- |
| *Streptomyces scabiei* 87-22 | FN554889.1 |
| *Streptomyces neyagawaensis* ATCC 27449 | DQ149987.1 |
| *Streptomyces stelliscabiei* DSM-41803 | JADBGF010000001.1 |
| *Streptomyces brasiliscabiei* IBSBF2867 | JABRXD010000015.1 |
| *Streptomyces griseiscabiei* NRRL-B-2795 | JAGJBZ010000002.1 |
| *Streptomyces graminofaciens* A-8890 | AP018448.1 |
| *Streptomyces humidus* JCM-4386 | BMTL01000005.1 |
| *Streptomyces* sp. JV178 | PEKU01000006.1 |
| *Streptomyces* sp. VNUA24 | CP119143.1 |
| *Streptomyces* sp. AM 4-1-1 | CP119145.1 |
| *Streptomyces* sp. AK08-02 | JARAZV010000001.1 |
| *Streptomyces* sp. B21-083 | JARUND010000002.1 |
| *Streptomyces* sp. NPDC008159 | JBIBHH010000016.1 |
| *Streptomyces* sp. NBC_01538 | CP109391.1 |
| *Streptomyces* sp. NPDC001351 | JBIAWS010000009.1 |
| *Streptomyces* sp. NPDC101112 | JBIVZL010000011.1 |
